# Direct cardiac effects of SGLT2 inhibitors

**DOI:** 10.1186/s12933-022-01480-1

**Published:** 2022-03-18

**Authors:** Sha Chen, Ruben Coronel, Markus W. Hollmann, Nina C. Weber, Coert J. Zuurbier

**Affiliations:** 1 Department of Anaesthesiology, Laboratory of Experimental Intensive Care and Anaesthesiology (L.E.I.C.A.), Amsterdam UMC, Location Academic Medical Centre (AMC), Amsterdam, University of Amsterdam, Cardiovascular Sciences, Meibergdreef 11, Room M0-129, Amsterdam, Noord-Holland 1105 AZ The Netherlands; 2 Department of Experimental Cardiology, Amsterdam UMC, Location Academic Medical Centre (AMC), Amsterdam,, University of Amsterdam, Cardiovascular Sciences, Amsterdam, The Netherlands

**Keywords:** Sodium-glucose-cotransporter 2 inhibitors, Ion homeostasis, Oxidative stress, Inflammation, Metabolism, Cardiac function

## Abstract

Sodium-glucose-cotransporter 2 inhibitors (SGLT2is) demonstrate large cardiovascular benefit in both diabetic and non-diabetic, acute and chronic heart failure patients. These inhibitors have on-target (SGLT2 inhibition in the kidney) and off-target effects that likely both contribute to the reported cardiovascular benefit. Here we review the literature on direct effects of SGLT2is on various cardiac cells and derive at an unifying working hypothesis. SGLT2is acutely and directly (1) inhibit cardiac sodium transporters and alter ion homeostasis, (2) reduce inflammation and oxidative stress, (3) influence metabolism, and (4) improve cardiac function. We postulate that cardiac benefit modulated by SGLT2i’s can be commonly attributed to their inhibition of sodium-loaders in the plasma membrane (NHE-1, Nav1.5, SGLT) affecting intracellular sodium-homeostasis (the sodium-interactome), thereby providing a unifying view on the various effects reported in separate studies. The SGLT2is effects are most apparent when cells or hearts are subjected to pathological conditions (reactive oxygen species, inflammation, acidosis, hypoxia, high saturated fatty acids, hypertension, hyperglycemia, and heart failure sympathetic stimulation) that are known to prime these plasmalemmal sodium-loaders. In conclusion, the cardiac sodium-interactome provides a unifying testable working hypothesis and a possible, at least partly, explanation to the clinical benefits of SGLT2is observed in the diseased patient.

## Introduction

Sodium-glucose co-transporter 2 inhibitors (SGLT2is) are now known to reduce the combined risk of cardiovascular death or hospitalization for heart failure in patients with heart failure with or without diabetes [1, 2]. SGLT2is were developed as an anti-diabetic drug, to inhibit the sodium-glucose co-transporter 2 (SGLT2) within the proximal tubule of the kidney. The SGLT2 is mainly present in epithelial cells within the proximal convoluted tubule, facilitating 90% of kidney glucose reabsorption from the glomerular filtrate. These on-target effects of SGLT2i result in improved metabolic, hormonal, and haemodynamic whole-body homeostasis that contribute to its cardiovascular benefits observed in large clinical trials. However, it seems unlikely that these kidney-mediated effects can explain the full spectrum of benefits, since previous trials with similar effects on glucose homeostasis or blood pressure are without such large and acute [3] beneficial effects on cardiac pathology observed with SGLT2is.

SGLT2is also have important off-target effects on isolated cardiomyocytes [4, 5], a cell-type that is commonly without SGLT2 [6]. These observations have initiated a plethora of studies uncovering direct SGLT2is effects on various cardiac cells, such as cardiomyocytes, endothelial cells, fibroblasts, smooth muscle cells, all of which largely devoid of SGLT2. It was soon recognized early that many of these off-target effects on cardiac cells target pathogenic mechanisms underlying heart failure (HF) and diabetic cardiomyopathy, and may thus contribute to the reported cardiovascular effects of these drugs [7–9]. For these diseases, the direct cardiac effects of SGLT2is constitute “on target” pharmacologic mechanisms. To avoid confusion, we will not use the terms on- and off target henceforward.

Here, we provide an up-to-date literature review summarising the current knowledge of the direct effects of SGLT2is on ion homeostasis, oxidative stress and inflammation, cell proliferation, fibrosis, energetic status and metabolism of cardiomyocytes, endothelial cells, smooth muscle cells, fibroblasts, platelets, and isolated hearts. It becomes apparent that SGLT2i in various cardiac cells modulates many of the important underlying cellular mechanisms that have been documented to contribute to cardiac pathologies such as hypertrophy, heart failure, diastolic dysfunction, and arrhythmias. We propose a testable working hypothesis that all these divergent SGLT2is effects are interdependent and can be integrated into a so-called cardiac sodium interactome.

## Literature search

**Fig. 1:**
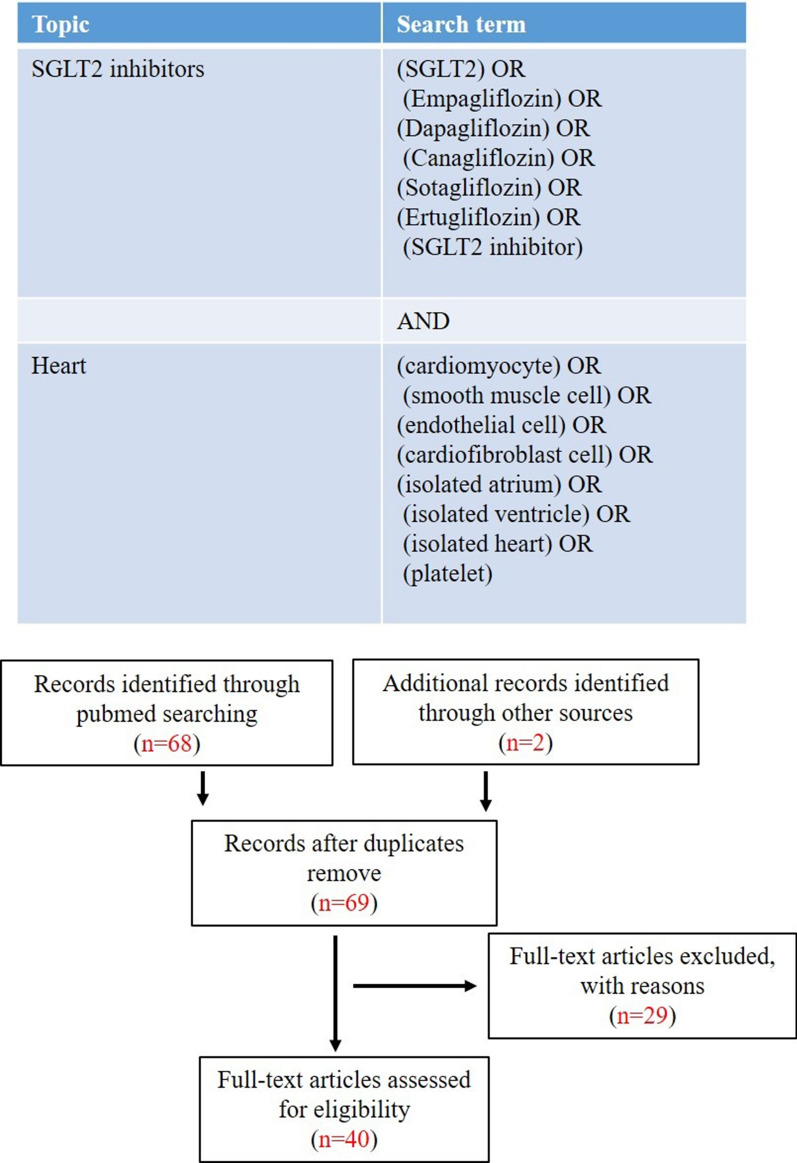
Search strategy and literature obtained. **A** Full search strategy with all search terms. **B** articles obtained and excluded. A total of 70 studies were initially retrieved from electronic databases using the search terms. There were 69 studies remaining after exclusion of duplicates using EndNote X9. There were 29 studies excluded because they did not study direct cardiac effects or applied overdoses of SGLT2i or contained not original research. In total we include 40 articles

We performed a literature search according to PRISMA guidelines [10] in Pubmed from 1975 until and including October 2021. The eligibility criterium was that studies had documented direct SGLT2is’ effects on cardiomyocytes, endothelial cells, smooth muscle cells, fibroblasts, platelets, and isolated hearts, as reported in Fig. 1. Exclusion criteria were studies employing clinically unrealistically high doses of SGLT2is (empagliflozin (EMPA), dapagliflozin (DAPA) and sotagliflozin (SOTA) > 1 μM; canagliflozin (CANA) > 3 μM). A total of 70 studies were initially retrieved from electronic databases. Sixty-nine studies remained after exclusion of duplicates using EndNote X9. Twenty-nine studies were excluded because of non-compliance with the eligibility criteria: direct cardiac effects were not examined (6 articles), [SGLT2is] is overdosed (8 articles), research was not original (15 articles). In total we included 40articles in this narrative review.

## Literature search results

### Ion homeostasis and ion transporters/exchangers

#### Cardiomyocytes and isolated hearts

Several reports have now demonstrated that SGLT2is affect Na^+^ and Ca^2+^ homeostasis in cardiomyocytes, although the effects are not consistently observed. Hamouda et al. [5] showed that 1 µM DAPA reduced systolic but not diastolic Ca^2+^ in isolated ventricular myocytes in the absence of albumin from streptozotocin (STZ)-induced diabetic mice. The effects were not observed in myocytes from healthy mice. We showed that acute (< 10 min) or more prolonged (3 h) 0.2–1.0 µM EMPA incubation in the absence of albumin lowered cytosolic Na^+^ in healthy rabbit cardiomyocytes [4]. Concomitantly, cytosolic Ca^2+^ was also reduced by EMPA. These effects were independent of the presence of glucose, indicating that effects were not mediated through SGLT1 or SGLT2. Further experiments demonstrated that EMPA directly inhibited the sodium-hydrogen exchanger (NHE)-1 in these rabbit cardiomyocytes. NHE-1 inhibition by EMPA was the mechanism responsible for lowering cytosolic Na^+^, because EMPA’s effects on Na^+^ were lost in the presence of the NHE-1 inhibitor Cariporide. Finally, EMPA, in the absence of albumin, acutely also increased mitochondrial Ca^2+^ in cultured electrically stimulated primary cardiac cells from rats. The effects on intracellular Na^+^ can be considered a class effect of SGLT2is, in that 1 µM EMPA, 1 µM DAPA and 3 µM CANA all reduced cytosolic Na^+^ and inhibited NHE-1 activity in the absence of albumin in healthy murine cardiomyocytes [11]. Further, Mustroph et al. [12] showed, in the absence of albumin, that 30 min or 24 h exposure to 1 µM EMPA significantly reduced cytosolic Na^+^ in isolated healthy murine cardiomyocytes, whereas 24 h EMPA (but not 30 min) exposure also reduced cytosolic Ca^2+^. EMPA treatment (15 min 1 µM) was also without effect on cytosolic Ca^2+^ in freshly isolated human heart failure ventricular cardiomyocytes [13].

The changes in ion homeostasis induced by SGLT2is also impact on cardiac electrophysiology. In myocytes from mice with heart failure or with a sodium channel mutation but not in healthy murine cardiac myocytes, 1 EMPA, in the absence of albumin, reduced late-I_Na_ in cardiomyocytes [14]. EMPA, DAPA and CANA were all potent and selective inhibitors of hydrogen peroxide (H_2_O_2_)-induced late-I_Na_ with little effect on peak-I_Na_. A reduction in late-I_Na_ contributes to less prolongation of the cardiac action potential and may protect against arrhythmias associated with prolonged action potentials [14].

Various studies have demonstrated that SGLT2is reduce cytosolic Ca^2+^ in cardiomyocytes under different conditions: EMPA (1 μM) for 2 h reduced lipopolysaccharide (LPS)-increased cytosolic Ca^2+^ in a human cardiomyocyte derived cell line in the absence of albumin [15]; 10–500 nM EMPA reduced doxorubicin (DOXO)-increased cytosolic Ca^2+^ in cultured HL-1 adult mouse cardiomyocytes in the presence of albumin [16]; However, not all studies reported effects on ion homeostasis: no decrease was observed in cytosolic Ca^2+^ or Na^+^ following short treatment (> 5 min) with DAPA (1 μM) in isolated cardiomyocytes in the absence of albumin from high-salt treated Dahl-sensitive rats [17], and no effects of EMPA (0.5 μM) on Ca^2+^ transient, sarcoplasmic Ca^2+^ load and diastolic sarcoplasmic Ca^2+^ leak in hiPSC-CMs in the absence of albumin were observed [18].

The inhibitory effect on NHE-1 by EMPA has recently been confirmed for human atrial cells and mouse ventricular myocytes in the absence of albumin [19]. However, Chung et al. [20] were unable to detect NHE-1 inhibition or reductions in cytosolic Na^+^ level in isolated rat ventricular cardiomyocytes by EMPA (1 µM) in the absence of albumin. Subsequent work by our group [21] demonstrated that some obvious methodological differences (buffer composition, external pH, pacing, % dimethylsulfoxide) between the studies [4, 11, 20] were unable to fully explain these divergent effects of SGLT2is, although there seemed to be a non-significant trend for loss of NHE-1 inhibition by SGLT2i at higher external pH [21].

In concordance with the lowering of intracellular calcium by SGLT2is, EMPA (1 μM) caused delayed cardiac contracture development during ischaemia in an NHE-1 dependent fashion in isolated mouse hearts in the absence of albumin subjected to ischaemia–reperfusion. However, EMPA was unable to mimic infarct size reduction during reperfusion by the NHE-1 inhibitor Cariporide, suggesting less potent NHE-1 inhibition by SGLT2is during the early reperfusion period than specific NHE-1 inhibitors [22]. However, Chung et al. was unable to observe lower cytosolic Na^+^ or impairment in NHE-1 activity with EMPA in isolated perfused healthy hearts of mouse, rat and guinea pig by Na triple quantum filtered nuclear magnetic resonance (NMR) and ^31^P NMR, respectively [20].

#### Endothelial cells

Our group recently demonstrated that EMPA (1 μM) also inhibited the NHE-1 activity and acutely lowered cytosolic Na^+^ in both human arterial coronary endothelial cells (HCAECs) and human umbilical vein endothelial cells (HUVECs) in the absence of albumin [23], and that EMPA (1 μM) reduced stretch-induced reactive oxygen species (ROS) production in HCAEC in the presence of albumin, at least partly through NHE-1 inhibition [24]. NHE inhibition by DAPA (1 μM) in the absence of albumin was also observed by Cappettta et al.in HUVECs [17].

#### Cardiofibroblasts

In cardiofibroblasts, 16 h incubation with DAPA (0.4 μM) decreased the LPS-induced increase in NHE-1 mRNA. Interestingly, on a protein level, DAPA specifically reduced the binding of NHE-1 to heat shock protein 70 (Hsp70), without affecting total protein levels of NHE-1 [25].

#### Platelets

Spigoni et al. [26] demonstrated that 15 min pre-incubation with EMPA (1 μM) or DAPA (1 μM) in the presence of albumin impaired activation of human platelet in an NHE-1 dependent fashion. Lescano et al. [27] also demonstrated that SGLT2i reduce human platelet activation in the presence of albumin, especially in the presence of endothelial-derived factors such as nitric oxide (NO) and prostaglandins. Both studies were unable to detect any SGLT2 mRNA or protein in platelets.

In summary, most studies support that SGLT2i inhibit NHE-1 and late-I_Na_ at the plasma membrane with downstream effects of decreased cytosolic Na^+^ and modulation of cytosolic and mitochondrial Ca^2+^ in cardiac cells. Given that not all studies confirm these effects of SGLT2is indicates that some modulatory factors are still unknown. These factors likely are related with pathological conditions, as most often, but not always, the direct cardiac effects of SGLT2is were observed during cellular stress. We speculate that oxidative, inflammatory, acid/base or mechanical stress may influence the results.

### Oxidative stress and inflammation

#### Cardiomyocytes and isolated hearts

Oxidative stress and inflammation both contribute significantly and interdependently to almost all major cardiovascular diseases. Inflammation can directly or indirectly induce acute or chronic oxidative stress through various cellular signalling pathways involving mediators as cytosolic protein kinase C (PKC) and calcium which activate ROS sources such as nicotinamide adenine dinucleotide phosphate oxidase (NOX) and the mitochondrial electron transport chain. Vice versa, oxidative stress is also a strong modulator of the inflammatory response through ROS-induced expressions of NLR family pyrin domain containing 3 (NLRP3) inflammasome and nuclear factor-kappa B (NF-kB) [28–30]. Because of this convoluted nature of oxidative stress with inflammation, effects of SGLT2is on these processes are simultaneously summarised and discussed.

In cardiomyocytes isolated from high-fat diet treated animals, EMPA (1 µM) for 2 h in the absence of albumin significantly restored low levels of Sestrin2 levels and AMP-activated protein kinase (AMPK) phosphorylation [31]. However, in cardiomyocytes isolated from standard chow treated animals, sestrin2 and AMPK phosphorylation were not decreased, probably explaining why EMPA in these conditions was unable to increase Sestrin2 and AMPK phosphorylation. The EMPA-induced AMPK phosphorylation enhanced the antioxidant nuclear factor erythroid 2-related factor 2 (Nrf2)/heme oxygenase-1 (HO-1) response in wild-type but not in Sestrin2 knock out mice. These data suggest that EMPA can only activate AMPK and reduce high-fat diet-induced ROS in metabolically stressed cardiomyocytes and does so through activation of sestrin2. In freshly isolated murine cardiomyocytes EMPA (0.5 μM) in the absence of albumin reduced hypoxia (H)/reoxygenation (R)-induced ROS [32]. This effect was partly dissipated by compound C, suggestive of ROS-reduction by EMPA being partially mediated through AMPK activation [32]. In addition, in HL-1 cardiomyocytes of mouse atrial origin, EMPA (1 μM) for 8 h in the presence of albumin was able to attenuate LPS-induced tumor necrosis factor alpha (TNFα) and inducible nitric oxide synthase (iNOS) expression [33]. Kolijn et al. [34] showed that 1 h treatment with EMPA (0.5 μM) in the absence of albumin of skinned fibers from LV biopsies of patients with preserved ejection fraction heart failure (HFpEF) significantly attenuated oxidative parameters and oxidized Protein kinase G Iα (PKGIα), resulting in improved phosphorylation of myofilament proteins and diminished cardiomyocytes stiffness, together with decreased levels of cytokines. Although these observations may bear relevance to HFpEF and the in vivo beneficial effects of EMPA, further research is necessary to substantiate that these effects were not mainly due to EMPA travelling directly into the cytosol of the skinned cardiomyocytes, because the plasma membrane was made permeable by the skinning process. Evidence is lacking that SGLT2i are taken up by intact cardiac cells.

In isolated mouse hearts pre-treated with EMPA (1 μM) in the absence of albumin and subjected to 30 min ischaemia and 40 min reperfusion, the expression of the innate immune receptor NLRP3, thioredoxin-interacting protein (TXNIP) and caspase-1 activity was blunted. In addition, EMPA reduced transcript levels of numerous markers of inflammation like interleukin (IL)-18, IL-1β, and TNFα. The ability of EMPA to prevent LPS-induced inflammation was completely inhibited by a Ca^2+^ ionophore. This suggested that EMPA attenuated LPS-induced NLRP3 inflammasome priming and cytokine production through lowering of Ca^2+^ [15]. Quagliariello et al. [16] demonstrated that 24 h incubation with EMPA (10–500 nM) in the presence of albumin reduced DOXO-induced IL-8, IL-6, IL1-β, leukotriene B4, myeloid differentiation factor 88 (Myd88), NLRP3 and p65/NF-kB expression in a HL-1 cell culture. In an acute model of myocardial injury, perfusion of isolated mouse hearts with 1 µM EMPA in the absence of albumin prevented activation of NLRP3 inflammasome and improved functional recovery after ischaemia [15].

In H9C2 cardiac cells, EMPA (1 μM) in the presence of albumin decreased the matrix metalloprotease (MMP)2 and MMP9 activity induced by IL-1 alpha and prevented apoptosis [35]. CANA (3 μM) in the presence of albumin was able to reduce ROS production in human right and left atrial tissue and cultured cells through inhibition of a SGLT1/AMPK/Rac1/NADPH oxidase pathway, whereas EMPA was without any effect [36].

#### Endothelial cells

Gaspari et al. [37] were the first to report direct endothelial effects of SGLT2i. They demonstrated that low concentrations of DAPA (< 5 nM) in the presence of albumin reduced TNFα-induced mRNA expression of NF-κB in HUVECs. Another study demonstrated that incubation with clinically relevant concentrations of CANA for 15 min, but not EMPA or DAPA, activated AMPK and inhibited IL-1β-stimulated adhesion of pro-monocytic U937 cells and secretion of IL-6 and monocyte chemoattractant protein-1 (MCP-1) in cultured human aortic endothelial cells (HAECs) [38]. Low-dose EMPA (100 nM) effects in the presence of albumin on hyperglycaemia-induced senescence of native and freshly isolated porcine coronary artery endothelial cells, show that EMPA reduced high glucose-induced ROS production and restored eNOS expression and NO formation through prevention of the activation of the endogenous angiotensin (Ang) system [39]. Further experiments demonstrated that high glucose induced SGLT1/2 expression in endothelial cells was prevented by EMPA, with EMPA also reducing glucose uptake in these hyperglycaemic conditions. EMPA was without effect on glucose uptake in normoglycaemic treated endothelial cells. Two-hour pre-treatment with 1 μM EMPA in the presence of albumin restored the beneficial effect of isolated cardiac microvascular endothelial cells (CMECs) on cardiomyocytes contraction and relaxation during CMEC incubation with TNFα [40]. EMPA in the presence of albumin reduced TNFα-induced mitochondrial and cytoplasmic ROS accumulation, which led to reinstatement of CMEC-derived NO delivery. No changes in eNOS expression or phosphorylation were observed. In a follow-up study of this same group [41], 1 μM EMPA for 2 h also reduced uraemic serum-induced ROS production in CMECs, an effect that could for ~ 30% be explained by NHE-1 inhibition. Ortega et al. [42] demonstrated that 24 h EMPA (0.3 μM) pre-incubation in the presence of albumin inhibited the activation of Ang II-induced p38/ mitogen-activated protein kinase (MAPK) and p65/NF-kB signalling pathways in HAECs. Our group also demonstrated restored NO formation together with reduced ROS production by EMPA and DAPA in the presence of albumin in TNFα-treated HCAECs, without alterations in eNOS expression [43].

Twenty four-hour pre-treatments with EMPA (500 nM) in the presence of albumin also reduced ROS produced during 1 h hypoxia and 3 h R in human microvascular endothelial cells (HMEC) [44]. Further experiments demonstrated that EMPA’s activation of signal transducer and activator of transcription 3 (STAT3) was downstream of EMPA’s ROS reduction, thus ROS reduction by EMPA was not through STAT3.

SGLT2i in the presence of albumin also reduced stretch-induced ROS production in HCAECs, an effect that was totally obliterated in the presence of the NHE-1 inhibitor Cariporide or a NOX inhibitor [24]. In addition, how inflammation induces ROS production in endothelial cells, and through what mechanism SGLT2is can prevent this, was recently reported [25]. ROS produced by the inflammatory cytokine TNFα in both HUVEC and HCEAC was caused by TNFα-induced activation of the NHE-1 and consequently increases in intracellular Na^+^. EMPA (1 μM) in the presence of albumin prevented the inflammation-induced ROS through direct NHE-1 inhibition and lowering of intracellular sodium. This study was the first showing the important role of changes in intracellular sodium in the regulation of oxidative stress in endothelial cells [23].

In freshly isolated endothelial cells from porcine coronary arteries, Ang II dose-dependently increased ROS production, an effect that was abolished by EMPA (0.1 μM) or SOTA (0.1 μM) in the presence of albumin [45]. In addition, Ang II or H_2_O_2_ increased the expression of SGLT1 and SGLT2. Prevention of increase in SGLT2 expression by siRNA, or by using EMPA or SOTA, decreased ROS-production by Ang II. Finally, ROS production by Ang II was also largely dissipated by removing glucose or Na from the medium, or by inhibition of NHE-1 or NCX, but not by NKA inhibition.

#### Smooth muscle cells

In cultured human aortic smooth muscle cells, EMPA (1 μM) in the presence of albumin pre-treatment reduced the pro-inflammatory IL-17α-induced oxidative stress, NLRP3 expression, caspase-1 activation, IL-1β and IL-18 secretion, partly through inhibition of SGLT2 [46]. These authors reported the presence of SGLT2 in these smooth muscle cells, and that SGLT2 expression was increased with IL-17α.

#### Cardiofibroblasts

In 3 h LPS-treated cultured freshly isolated cardiofibroblasts from healthy C57BL/6 J mouse hearts, 16 h pre-incubation with DAPA, becoming effective at a concentration of 0.3 μM, reduced expression of NRLP3, TNFα and caspase-1 [47]. The effect was AMPK-dependent as P-AMPK/total-AMPK ratio was significantly increased after DAPA treatment, and inhibition of AMPK activation abolished DAPA’s effects.

In summary, applying a plethora of various pathological conditions (high-fat diet, H/R, ischemia/reperfusion, LPS, DOXO, Il-1α, IL-1β, TNFα, IL-17α, Ang II, hyperglycaemia, or an oxidative stress generator) most literature clearly indicates that SGLT2is directly reduce inflammation and/or oxidative stress in various cell types under these pathological conditions. In healthy cells, anti-inflammatory/oxidative stress effects of SGLT2is are less prominent. To which extent the stress-dependency of SGLT2i’s effects is explained by stress-induced expression of SGLT1/2 and/or stress-activated NHE-1/late-I_Na_ has not been resolved and constitute an important quest for future research.

### Metabolism

#### Cardiomyocytes or isolated hearts

Direct metabolic effects of SGLT2is on cardiac cells showed that 1 μM EMPA for 24 h in the presence of albumin increased expression of glucose transporter (GLUT) 1, but not GLUT4, in isolated quiescent cardiomyocytes from human end-stage HF transplanted hearts or healthy and transverse aortic constriction (TAC)-induced HF murine hearts [48]. EMPA also increased 2-deoxyglucose uptake in the TAC heart isolated cardiomyocytes, following a starvation period without glucose being present. To what extent these findings can be extrapolated to the intact beating heart supplied with a mixture of substrates is unclear, also considering that approximately 50% of the examined isolated cells had already died [48]. Although several studies report changes in cardiac metabolism following in vivo chronic SGLT2i treatment, these studies provide no information on direct cardiac effects of SGLT2i because (1) metabolism is altered due to systemic changes in substrates and hormones because of glucosuria in the kidney, and (2) an SGLT2i was not present in the isolated heart model. Few studies have examined direct cardiac metabolic effects of SGLT2i. In isolated type 2 diabetic mice hearts, metabolic effects of 35 min EMPA (1 μM) perfusion in the presence of albumin on ^13^C-glucose or ^13^C-palmitate metabolism have been examined [49]. Although EMPA was without an overall effect on the major metabolic pathways of glucose or fatty acid metabolism, EMPA did reduce lactate labeling from ^13^C glucose, and increased α-ketoglutarate labelling from ^13^C palmitate. In a separate series of experiments, EMPA effects on lactate labelling were dissipated in the presence of the NHE-1 inhibitor Cariporide, suggesting that these direct metabolic effects were mediated through NHE-1 inhibition [45].

Overall, it appears that direct metabolic effects of SGLT2i on the heart may only mildly shift metabolism away from glucose-use towards fatty acid oxidation. However, studies that examine direct cardiac effects of SGLT2is for longer periods than 1 h and on diseased hearts are anticipated. In these models the shift in metabolism may be demonstrated more clearly.

### Cardiac function

The ex vivo isolated perfused heart is an ideal model to examine direct cardiac effects of SGLT2i, because during SGLT2i treatment cardiac function of the intact beating heart can be monitored without alterations in blood pressure, coronary flow, or changes in substrates or hormones delivered to the heart. This is not possible in the in vivo condition because of the strong effect SGLT2i has on the kidney resulting in acute diuresis, natriuresis and glycosuria. During normoxic perfusion with a balanced physiological mixture of the important substrates for the heart, 1–3 μM SGLT2i in the absence or presence of albumin was without effect on any of the cardiac performance parameters of the healthy or type II diabetic mouse hearts [11, 22, 49]. In line with the supposed action of SGLT2is on the NHE, the NHE-1 inhibitor Cariporide was also without any effect on cardiac performance [22]. The absence of effects fits the idea that NHE-1 activity is low during normoxic pH-neutral (pH = 7.4) conditions as present in non-pathologic hearts. However, during ischaemic conditions SGLT2is did influence cardiac performance: delaying contracture development during total global ischemia or preservation of contractile function during regional ischaemia [22, 50]. Subsequently, following the ischaemic insult, most studies have reported improvements in cardiac function in the presence of 1 μM EMPA [14, 51].

It thus seems that SGLT2i, although without effects on cardiac function in the healthy heart, can improve cardiac function during ischaemic and reperfused conditions, again demonstrating that direct cardiac effects of SGLT2i mainly become apparent in the diseased heart.

## Discussion

SGLT2is have changed the therapeutic field of diabetes and heart failure, due to the consistent cardiac and renal protective effects as reported in several large clinical trials [1, 2]. A recent meta-analysis, including eleven cardiovascular outcome trials (CVOTs) with more than 75,000 patients, concluded that treatment with SGLT2is in patients with cardiometabolic and renal diseases resulted in a sustained to moderate reduction of the composite cardiovascular death or hospitalization for HF, robust reduction of HF, and a moderate reduction of cardiovascular mortality, total mortality and major cardiovascular events [52]. Although the benefit of SGLT-2 inhibitors surpass that obtained with glucagon-like peptide-1 receptor agonists (GLP-1RA), it does seem that the gap between the cardiac benefits of SGLT-2 and GLP-1RA is narrowing [53, 54].It is commonly accepted that the large beneficial clinical effects cannot be explained by one single mechanism targeted by SGLT2is. Most likely the underlying mechanisms are related to both SGLT2 on-target kidney effects and to non-kidney, non-SGLT2 off-target effects. The kidney on-target effects consist of glycosuria, diuresis, natriuresis and improved kidney function that precipitate into systemic effects as blood pressure lowering, improved glucose/insulin balance, increased erythropoietin(EPO), improved crosstalk between kidney and heart, fluid and Na redistribution between specific body compartments, and a shift towards a more fasting whole-body state [8, 55, 56].

**Fig. 2:**
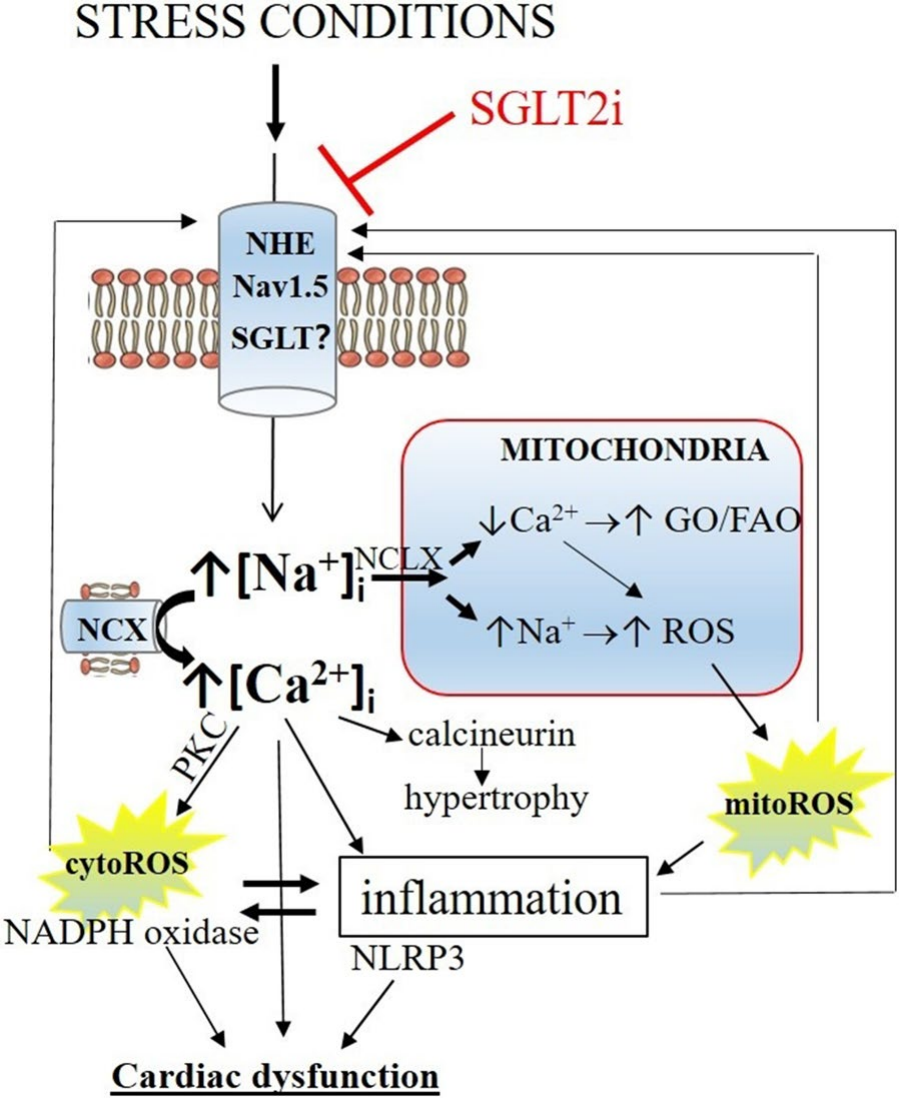
SGLT2i and the Sodium-interactome of cardiac cells. Stress conditions will activate the sodium/hydrogen exchanger (NHE) and/or sodium channel (Nav1.5) and/or induce expression of SGLT2 in the plasma membrane facilitating increases in cytosolic sodium ([Na^+^]_I_. Increases in ([Na^+^]_I_ can result in increased calcium ([Ca^2+^]_I_ through impairment of NCX. ([Na^+^]_I_ will transfer into mitochondria through the sodium/calcium exchanger (NCLX) in the mitochondrial membrane, increasing mitochondrial Na^+^ and decreasing mitochondrial Ca^2+^. Decreased mitochondrial Ca^2+^ shifts metabolism away from fatty acid oxidation (FAO) towards glucose oxidation (GO) and facilitates ROS production through diminished anti-oxidant activity of ROS detoxifying enzyme systems. Increased mitochondrial Na^+^ can increase mitochondrial ROS through decreases in membrane fluidity. Increased cytosolic Ca^2+^ stimulates hypertrophy through calcineurin activation, cytosolic ROS production through activation of NADPH oxidase, and inflammation through activation of the Nlrp3 inflammasome. Oxidative stress and inflammation causes cardiac dysfunction and positively feed backs to the NHE and Nav1.5 by activating these channels

In this review we summarize the major experimental findings related to so-called off-target and direct cardiac effects of SGLT2is. It becomes apparent that SGLT2i in various cardiac cells modulates many of the important underlying cellular mechanisms that have been documented to contribute to cardiac pathologies such as hypertrophy, heart failure, diastolic dysfunction, and arrhythmias. Especially during treatment with disease-associated mediators such as inflammatory (cytokines, LPS) and metabolic stressors (hyperglycaemia, high-fat, low oxygen, ischemia, acidosis), SGLT2is were able to reduce the imposed pathological disturbances in physiological entities such as ion homeostasis, redox status, metabolism, and cardiac function. It is unlikely that SGLT2i affects all these entities separately and independently of each other. We propose that these SGLT2i-sensitive entities are all connected through a common nodal point that is affected by SGLT2is, i.e. the cellular interactome with [Na^+^]_i_ as the common nodal point (Fig. 2). Below we discuss how the reported SGLT2is effects on one system, as provided by the review, can propagate to affect the other entities, thereby redirecting the entire physiological syncytium away from a cardiac pathological destination towards a more healthy, sustainable cardiac condition.

### Working hypothesis: Intracellular [Na^+^]_i_ as nodal point of the cardiac interactome affected by direct cardiac effects of SGLT2is

Almost all cells in the body have a large sodium gradient over the cell membrane that is maintained by the energy-demanding Na^+^/K^+^ pump, with intracellular concentrations around 10 mM, and extracellular concentrations between 135–145 mM. The sodium gradient is necessary for various essential physiological processes ranging from the creation of an action potential in excitable cells towards the facilitation of cellular exchange of various molecules through Na-cotransporters/exchangers. Changes in [Na^+^]_i_ can act as a critical second messenger which regulates many cellular functions. It has direct consequences for e.g. [Ca^2+^]_i,_ oxidative stress, immunity/inflammation regulation and mitochondrial function. This interactome of [Na^+^]_i_ in relation to potential effects of SGLT2is is schematically depicted in Fig. 1.

Elevated intracellular sodium is a hallmark of the failing and diabetic heart, and the increases in [Na^+^]_i_ with mechanical or metabolic overload have been ascribed to increased NHE activity, late-I_Na_, NCX or SGLT1/2 [57–62]. Increases in [Na^+^]_i_ can trigger multiple downstream signalling pathways known to be active in the failing heart. Elevated [Na^+^]_i_ causes an increase of mitochondrial Na^+^ at the expense of mitochondrial Ca^2+^ through the mitochondrial sodium-calcium exchanger (NCLX). Increases in mitochondrial Na^+^ reduces the fluidity of the mitochondrial inner membrane thereby promoting mitochondrial ROS production [63]. The decreased mitochondrial Ca^2+^ hampers the tricarboxylic acid (TCA)-derived nicotinamide adenine dinucleotide (NADH) generation through inhibition of dehydrogenases or inhibit F_0_F_1_ Adenosine triphosphate (ATP) synthase, resulting in energy deficits and reduced nicotinamide adenine dinucleotide phosphate (NADPH) generation for antioxidant activity [64]. In addition, elevated cytosolic Na^+^ in the heart has been shown to cause a shift in cardiac metabolism away from fatty acid metabolism towards glucose metabolism through the lowering of mitochondrial Ca^2+^ and/or increases in cytosolic Ca^2+^ [65, 66]. Energy deficiency, decreased oxidative capacity, increased oxidative stress, increased glycolysis and attenuated fatty acid metabolism are all characteristics of the hypertension/post- myocardial infarction (MI) induced failing hearts [67–69]. The decreased oxidative capacity is likely not due to oxygen limitation or ischemia but to metabolic remodelling, as oxygen limitation has not been observed in failing hearts [70–72]. Subsequently, cytosolic Ca^2+^ increases due to inhibition or partial reversal of the sarcolemmal NCX. Increases in [Ca^2+^]_i_ can then drive cardiac hypertrophy through activation of calcium-dependent phosphatase calcineurin [73]. Elevated [Ca^2+^]_i_ also activates NADPH oxidases to produce ROS through PKC activation [74]. Increases in [Ca^2+^]_i_ and cytosolic and mitochondrial ROS can activate the NLRP3 inflammasome to convert pro-IL-1β into the pro-inflammatory IL-1β [75], which upon release from the cell by pyroptosis can induce general inflammatory mediators such as IL-6 and TNFα. The produced ROS and inflammatory mediators can then activate the sarcolemmal NHE-1 or late-I_Na_ to further contribute to increase in [Na^+^]_i_ [23, 76]. The oxidative stress can also oxidize the Ca^2+^/Calmodulin-dependent kinase IIδ (CaMKII) that then augments the late-I_Na_ and activates NHE-1, leading to further Na^+^ and Ca^2+^ overload [77, 78]. Increased cytoplasmatic Ca^2+^ also plays an important role for the genesis of life-threatening cardiac arrhythmias. Thus, we propose that in diseased states of the heart where inflammation, oxidative stress, and Na^+^ and Ca^2+^ overload are present, such as in heart failure syndromes, these mechanisms amplify each other in a positive feedback loop which propagate the pathology. The present review demonstrates that SGLT2i reduces many of the mechanisms directly within the various cardiac cells. We propose the working hypothesis that SGLT2i puts a brake to the positive feedback loop activated in diseased states through inhibition of Na^+^-loading transporters in the cell membrane.

### Controversies in reported direct cardiac effects of SGLT2i

For several variables which have been evaluated, ambivalence exists in whether the variable is changed by SGLT2i. The discrepancies between laboratories are possibly due to differences in experimental conditions and/or models that determine whether (1) the suggested SGLT2i-targets (NHE-1, Nav1.5, SGLT1 or 2) are present and active, (2) the suggested outcome variables (NO, AMP, Na^+^, Ca^2+^, ROS) can be modulated, and (3) the applied methods are sensitive enough to detect small changes in the variable.

This is e.g., illustrated by the interaction between SGLT2i and the sodium channel Nav1.5, which facilitates the late-I_Na_: only when Nav1.5 becomes oxidized can SGLT2i affect this channel [14]. There is also a tendency for SGLT2i’s effects on NHE-1 activity to become more pronounced at lower external pH [21], thus with acidification. The increased sensitivity of NHE-1 with lowering of pH for inhibition by SGLT2i may also explain why NHE-1 inhibition by EMPA was only observed in the presence of an inhibitor of the sodium/potassium pump [79], inhibition of which is known to cause acidosis. A recent article demonstrated direct binding of DAPA to the NHE-1 using surface plasmon resonance techniques [80]. In addition, NHE has low activity in normal healthy myocardium but much higher activity in e.g., failing myocardium, such that sodium lowering through NHE inhibition (or SGLT2is) is much more prominent and easier to detect in cardiomyocytes isolated from HF versus healthy hearts [58]. Finally, recent research indicates that pathological conditions may stimulate the expression of SGLT2, such that even in heart tissue SGLT2 becomes present. Increases in Ang II and oxidative stress increased SGLT2 expression in porcine coronary artery endothelia cells [45], post-MI induced activation of renal sympathetic activity and/or norepinephrine increased renal SGLT2 [81], and post-MI conditions transiently increased SGLT2 expression in the ischemic heart [82]. Glucose transport through the SGLT2 is co-transported by Na^+^, such that transport through SGLT2 will also result in increased [Na^+^]_i_. The findings from the current review that cells become more sensitive to SGLT2i treatment under stress conditions, underscores the importance of experimental conditions. Concerning sensitivity of variables to modulation, heterogeneity in metabolism between cells or models is an important determinant affecting the basal physiological condition and therefore sensitivity of the scrutinised biological system to perturbations instigated by SGLT2i treatments [75, 83]. For example, whether SGLT2i increase AMPK signalling in animal-derived cardiomyocytes depends on the nutritional treatment of the animals [32], and other factors that dictate the metabolic balance between glycolytic and mitochondrial activity and thereby AMPK’s sensitivity to modulation in cells [83]. SGLT2i effects on eNOS activity are also not always observed, which also could be related to differences in mechanical and metabolic conditions applied to the ECs in the separate studies. For example, hyperglycaemic or oscillating flow conditions induce O-GlcNAcylation of the eNOS enzyme, thereby preventing that increases in enzyme activity can occur [84]. Finally, differences in measurement sensitivity between research laboratories may also have its impact. For example, SGLT2i induced small decreases in [Na^+^]_i_ of < 2 mM at low absolute concentrations [4, 11] that cannot be measured by a standard fluorescence [Na^+^]_i_ assay. Measuring small changes in intracellular [Na^+^]_i_ at low [Na^+^]_i_ can be challenging and requires optimization of optical detection. We have developed such a specific and sensitive method using the fluorescence probe SBFI (sodium-binding benzofurzan isophthalate) [85]. Through optimisation of wavelengths, using a dual emission ratio mode (not dual excitation as is commonly used) and a narrowing window viewing surface of one myocyte only, has led to the detection of these small changes in Na^+^ by SGLT2i [85].

In summary, while there is still much to be understood about the various factors which determine how SGLT2i’s affect cardiac cells, most research clearly demonstrates that direct cardiac effects of these SGLT2i can be observed, especially in pathological conditions, and that the effects are mediated by altered intracellular sodium. Obviously, more research is needed here to further understand SGLT2i’s cellular mechanisms. It is hereby important to note that whereas cardiac effects of SGLT2i’s were not always observed in all studies, they were almost always observed under pathological, stressed, conditions. This makes these observed cardiac cellular effects highly relevant for the understanding of underlying mechanisms in the clinical condition where SGLT2i’s are only used in diseased patients.

### Limitations

The review summarizes research that examined direct and acute effects of SGLT2i on cardiac cells. This obviously deviates from chronic treatments in large clinical trials, and it is therefore not known to which degree the direct and acute cardiac effects described in this review persist in the chronic condition. However, recently studies have reported that SGLT2i also improve outcome in the acute phase of heart failure in patients [86] and EMPA-Response trial [87], suggesting that also clinically SGLT2i have acute beneficial effects.

In this review we only selected studies employing up to a maximal concentration of 1 μM for EMPA or DAPA. This concentration is close to the maximum concentration (bound + free SGLT2i) measured in human plasma when treated with 25 mg/kg/day EMPA or 10 mg/kg/day DAPA (Cmax between 0.4–1.1 μM) [88, 89]. Additionally, in vivo SGLT2i are largely (80–99%) bound to plasma proteins [89], whereas several articles discussed in this review performed measurements in the absence of any proteins. Thus, these articles have employed rather high concentration of free, unbound SGLT2i. Numerous articles in this review have only applied values close to this Cmax, which is a logical first step in the discovery of possible cellular effects of SGLT2i, especially when many different processes/signals are examined. However, now that many direct cellular effects of these drugs are discovered and established and knowing also that the plasma drug concentration during the day falls to below 20% of the Cmax [88, 89], a critical next step should be the full dose–effect relationship determination for the range of the free SGLT2i plasma concentrations observed in humans. It can even be argued that the association of the drug concentration and its effects can best be studied at the trough value, although uncertainty exists for this proposition. However, even when these SGLT2i direct cardiac effects are only present at values close to the clinical Cmax, this may still be clinically relevant when this would result in intermittent effects (2–6 h)) every day. In addition, to apply slightly higher concentrations in cell studies is a common approach to partly compensate for the much shorter duration of drug exposure (usually hours to days) in cellular or organismal studies compared to human applications of these drugs lasting for months to years. Finally, plasma concentration is not always an indicator of effect if the distribution volumes are compartmentalized, or when receptors are involved, as is likely the case with SGLT2i. Especially when studying isolated cardiomyocytes (as discussed in this review), in the in vivo condition these cells are in the interstitial compartment where the free unbound drug can accumulate on receptors during months-to-years of treatment, making it rather difficult to know with certainty what concentration is actually seen by these receptors. Nevertheless, further research is warranted to determine the full dose–response relationship (10–200 nM unbound SGLT2i) of many of the direct cardiac effects reported in this review, to further test the validity of the reported cardiac effects for the in vivo human condition.

## Conclusion

SGLT2is can have direct cardiac effects that all seems to be mediated by modulation of intracellular sodium concentration, the sodium-interactome. Further experiments in various ex vivo and in vivo conditions are needed to test and validate this working hypothesis that SGLT2i have direct cardiac effects through alteration of the sodium-interactome. The changes are likely an explanation, at least partly, for the clinical benefits of SGLT2i-treatment.

## Data Availability

Not applicable.
